# Patterns of Microbiome Composition Vary Across Spatial Scales in a Specialist Insect

**DOI:** 10.3389/fmicb.2022.898744

**Published:** 2022-06-02

**Authors:** Kyle J. Paddock, Deborah L. Finke, Kyung Seok Kim, Thomas W. Sappington, Bruce E. Hibbard

**Affiliations:** ^1^Division of Plant Science and Technology, University of Missouri, Columbia, MO, United States; ^2^Department of Natural Resource Ecology and Management, Iowa State University, Ames, IA, United States; ^3^USDA-ARS, Corn Insects and Crop Genetics Research Unit, Iowa State University, Ames, IA, United States; ^4^USDA-ARS, Plant Genetics Research Unit, University of Missouri, Columbia, MO, United States

**Keywords:** insect-microbe interactions, microbial ecology, biogeography, *Diabrotica virgifera virgifera*, beetle, metacommunity

## Abstract

Microbial communities associated with animals vary based on both intrinsic and extrinsic factors. Of many possible determinants affecting microbiome composition, host phylogeny, host diet, and local environment are the most important. How these factors interact across spatial scales is not well understood. Here, we seek to identify the main influences on microbiome composition in a specialist insect, the western corn rootworm (WCR; *Diabrotica virgifera virgifera*), by analyzing the bacterial communities of adults collected from their obligate host plant, corn (*Zea mays*), across several geographic locations and comparing the patterns in communities to its congeneric species, the northern corn rootworm (NCR; *Diabrotica barberi*). We found that bacterial communities of WCR and NCR shared a portion of their bacterial communities even when collected from disparate locations. However, within each species, the location of collection significantly influenced the composition of their microbiome. Correlations of geographic distance between sites with WCR bacterial community composition revealed different patterns at different spatial scales. Community similarity decreased with increased geographic distance at smaller spatial scales (~25 km between the nearest sites). At broad spatial scales (>200 km), community composition was not correlated with distances between sites, but instead reflected the historical invasion path of WCR across the United States. These results suggest bacterial communities are structured directly by dispersal dynamics at small, regional spatial scales, while landscape-level genetic or environmental differences may drive community composition across broad spatial scales in this specialist insect.

## Introduction

Animals have evolved while in constant contact with microorganisms. Associations between hosts and microorganisms exist on a continuum from beneficial to detrimental. In insects, bacterial communities can improve host fitness by enhancing nutrition (Ben-Yosef et al., 2014), disrupting plant defenses (Chu et al., 2013), and protecting against disease (Miller et al., 2021), but are also capable of inducing mortality in certain situations (Caccia et al., 2016; Mason et al., 2019). Variation in bacterial communities within hosts may result in differential survival with direct implications for design and implementation of conservation and pest management strategies (Paddock et al., 2021).

Inter- and intra-species variation in the microbiome can be influenced by numerous factors. Host species identity can dictate what microbes survive within the host. Insect guts vary in morphology, pH, and immune response (Caccia et al., 2019), which serve as filters for specific microbes resulting in communities that vary between closely related species (Adair et al., 2020). Host diet partially determines the local species pool with which the host interacts. Different feeding modalities (e.g., chewing vs. sucking mouthparts) constrain access to food sources, which can affect which microbes are associated with insects (Huang et al., 2021). Furthermore, certain food substrates can be digested by microbes within insects, and thus can alter communities through resource limitation or niche partitioning (Mason et al., 2020; Brochet et al., 2021). The host’s external environment can also generate variation between insect microbiomes by affecting the local source pool of microbes. Such factors as temperature (Wang et al., 2020), landscape context (Park et al., 2019), and plant diversity (Cohen et al., 2020) may be associated with variation between insect microbiomes in different local habitats.

Host microbiomes across a biogeographic space can best be described through a metacommunity framework (Adair and Douglas, 2017; Miller et al., 2018), where host-associated communities in local environments are subsets of the larger environmental metacommunity and linked through dispersal. Spatial limits on microbial dispersal, or “dispersal limitation,” can result in patterns of geospatial correlation in which microbial community similarity decays with increasing geographic distance (Finkel et al., 2012; Moeller et al., 2017). At different spatial scales, the determinants of microbiome composition may change and result in different communities. Both landscape composition and configuration can dictate the dispersal capacity of microorganisms within the metacommunity (Parajuli et al., 2020). For example, in humans, small-scale dispersal events may be disrupted by barriers such as human-made structures or vegetation (Parajuli et al., 2020). At a continental scale, dispersal may be very limited and other factors such as lifestyle, diet, age, and genetics may account for most variation between hosts (Yatsunenko et al., 2012). Which microbes are associated with hosts is determined by interactions of both deterministic and stochastic processes (Adair and Douglas, 2017), but how these processes interact across spatial scales is not well understood.

Corn rootworms (genus *Diabrotica*; Coleoptera, Chrysomelidae) represent a useful system to investigate the influence of biogeographical arrangement on host associated microbiome composition. Many studies have investigated determinants of microbiome composition in generalist feeding insects (Adair et al., 2018; Jones et al., 2019; Cohen et al., 2020). Studies with generalist insects have limitations on distinguishing between the influence of geographic location and the influence of diet on microbiome composition. In the United States, two rootworm species predominate east of the Rocky Mountains: the western corn rootworm (*Diabrotica virgifera virgifera* LeConte; WCR) and the northern corn rootworm (*Diabrotica barberi* Smith & Lawrence; NCR). Both species overlap in distribution, phenology, and host plant usage (Krysan and Miller, 1986), which allows us to better examine environmental factors influencing microbiome composition. In addition, they comprise the most damaging groups of corn pests in the United States, with management costs and yield losses combining for over $2 billion annually (Wechsler and Smith, 2018). Management continues to become more complicated as both species have evolved resistance to crop rotation and transgenic crops producing toxins derived from the bacterium *Bacillus thuringiensis* (Bt; Levine et al., 1992, 2002; Calles-Torrez et al., 2019). In WCR, the microbiome may help beetles overcome plant defenses, and changes in larval microbiome composition are linked to resistance to Bt (Chu et al., 2013; Paddock et al., 2021). The geospatial consistency of bacterial community composition within WCR has yet to be investigated.

Here, we characterize the bacterial communities in two sister *Diabrotica* species and compare the patterns of assembly at different spatial scales. We collected WCR beetles from corn fields across their ranges in the United States using two sampling scales, regional scale (~12–50 km between sites along linear transects) and continental scale (>200 km). We examined correlations between bacterial community dissimilarity and distance between collection sites for WCR. We hypothesize that environment contributes to variation in microbial communities, and thus, differentially influences the similarity of microbial communities across biogeographical space. Specifically, we predict that microbiome sequence similarity decreases with geographic distance (distance decay), and that the effect is stronger at the landscape level where dispersal limitation may be higher compared to local levels. Increased understanding of the factors influencing microbiome composition in insects can provide insight into how microbe-mediated effects on plant-insect interactions may have evolved.

## Materials and Methods

### Insects

**Figure 1:**
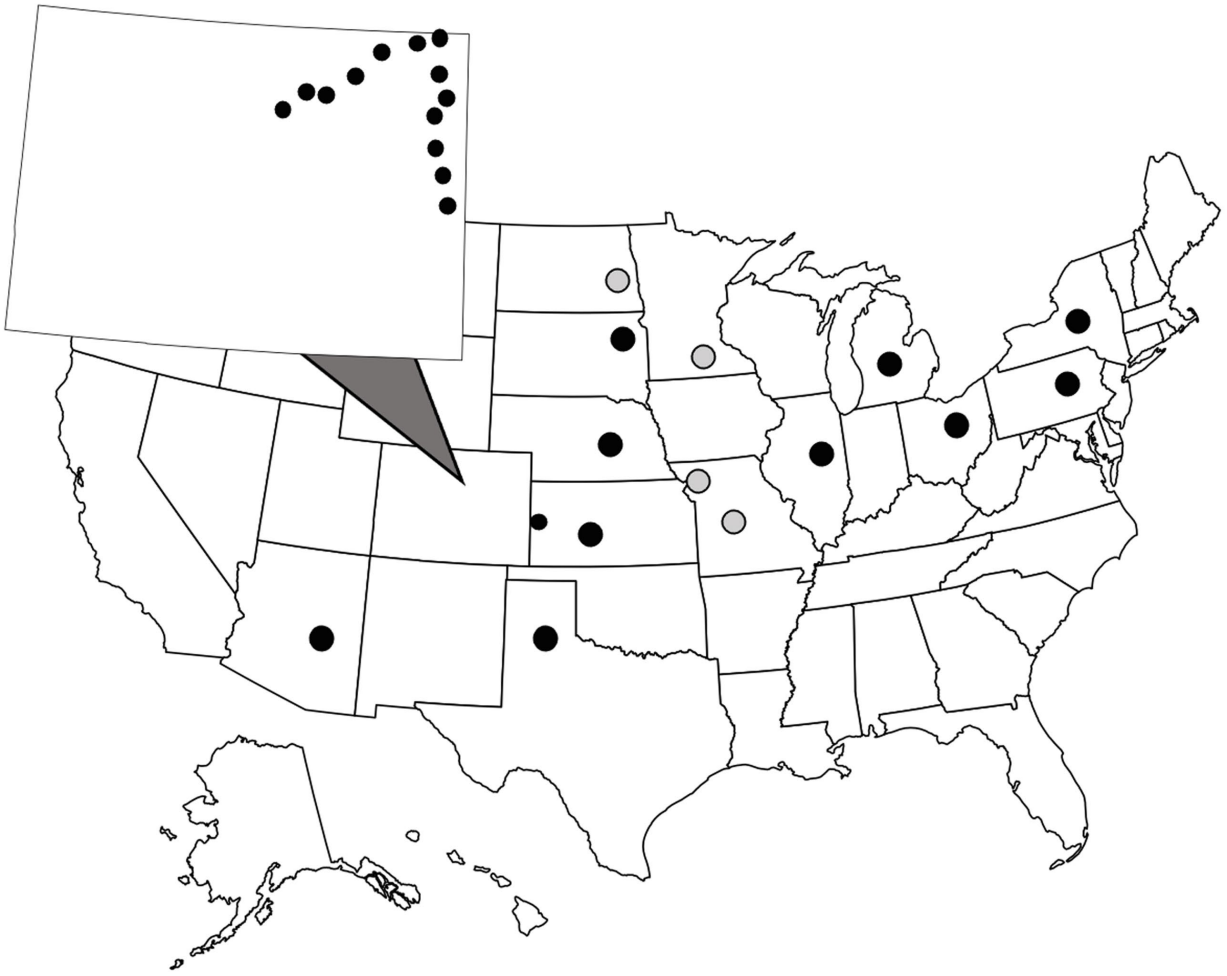
Sample locations of wild, adult *Diabrotica virgifera virgifera* (black dots) and *D. barberi* (gray dots). Beetles were collected from cornfields during late July to early August. Colorado is zoomed out with small circles showing close-proximity sampling locations (note one location is across the border in Kansas). Each dot consists of one sample site where eight individual beetles were collected.

WCR and NCR are univoltine species that emerge in late July into August. In this study, adult beetles were collected from corn fields between July and August in 2016, 2019, and 2020. Information including date of collection and location of the closest city or populated place to the collection site can be found in Supplementary Information. WCR were collected at two spatial scales (Figure 1). A small, regional-scale sampling scheme consisted of field sites located ~25 km apart along two transects in eastern Colorado and western Kansas. On average, WCR can disperse ~17 m a day. Distances between any two sites ranged from 11.46–276.73 km. Beetles were collected in ethanol and stored at −20°C at the Corn Insects and Crop Genetics Research Unit (CICGRU) in Ames, Iowa until DNA extraction. For the broad, continental-scale sampling scheme, field sites were ~250 km apart, with paired distances ranging from 248 to 3,122 km. Beetles were stored in 95% ethanol and shipped to Columbia, Missouri, where they remained until DNA was extracted. A total of 24 sites across 11 states were sampled for WCR in 2016, 2018, and 2020. Within the small regional-scale sampling scheme, 14 city level sample sites were collected in 2016. Eight beetles were collected at each site for a total of 192 WCR beetles. NCR were collected only at a continental scale and were processed in the same manner as WCR. A total of four sites were sampled for NCR in 2020. Within Missouri, two city level samples were collected. Eight beetles were collected at each site for a total of 32 NCR beetles.

### DNA Extraction and 16S rRNA Gene Amplification

For each sample location, DNA was extracted from individual beetles (*n* = 8). Before the day of extraction, beetles in ethanol were removed and placed in individual 2-ml tubes to dry overnight. Beetles were transferred to beaded tubes (MP Biomedicals, Santa Ana, CA, catalog no. 116913500), flash frozen in liquid nitrogen for 20 s, immediately placed in a single-tube bead beater, and shaken for 20 s to pulverize the sample. Bacterial DNA was extracted from pulverized beetles using PowerFecal® DNA Isolation Kit (QIAGEN, catalog no. 12830-50) in accordance with the manufacturer’s protocols.^1^ Frozen beetles processed at CICGRU were ground by mortar and pestle and then transferred to sterile 1.5-ml microcentrifuge tubes. Bacterial DNA was extracted using DNA Blood and Tissue kit (QIAGEN, catalog no. 69504) in accordance with the manufacturer’s protocols.^2^ The concentration of extracted DNA was quantified using a Qubit 2.0 fluorometer (Invitrogen, Carlsbad, CA). DNA was stored at −80°C until further downstream processing. Prior to amplification, DNA concentration was standardized to 3.51 ng/μl. The V4 hypervariable region of the 16S rRNA gene was amplified using single indexed universal primers (U515F/806R) with Illumina standard adapter sequences. Dual-indexed forward and reverse primers were used in all reactions. PCR reaction steps were as follows: 98°C^(3:00)^ + 25 cycles of [98°C^(0:15)^ + 50°C^(0:30)^ + 72°C^(0:30)^]. The resulting amplicons (5 μl) were pooled before sequencing on the Illumina MiSeq 2 × 250 bp platform (Paddock et al., 2021). The construction and sequencing of 16S sequencing amplicon libraries were completed at the University of Missouri (MU) DNA Core facility.

### 16S rRNA Community Analysis

Sequence assembly and annotation were performed by the MU Informatics Research Core Facility. Primers were trimmed using Cutadapt in two rounds,^3^ first removing forward primers with an error rate of 0.11 mismatches and minimum length of 19 bp, followed by a second round of trimming from the 3′ end executed with an error rate of 0.1 mismatches and minimum length of 20 bp. A minimal overlap of 3 with the 3′ end of the primer sequence was required for removal. Untrimmed contigs were discarded between rounds of trimming. Using the Qiime2 plugin, DADA2 (Callahan et al., 2016; version 1.10.0), forward and reverse reads were truncated to 150 bp and discarded if the number of expected errors was >2.0. Chimeras were detected using the “consensus” method and removed. Resulting sequences were filtered to retain only sequences 249–257 bases long. Taxonomy was assigned to amplicon sequence variants (ASVs) using the Silva.v132 database with the “sklearn” classifier in Qiime2. ASVs were compiled into biom tables for data analysis. ASV tables with metadata and associated taxonomy were imported in to RStudio version 3.5.2 for downstream analysis. ASVs matching chloroplast, mitochondria, and archaea sequences were filtered and removed using phyloseq::filter_taxa in RStudio (McMurdie and Holmes, 2013). Taxa labeled “uncharacterized” at the phylum level were also removed. An extraction blank was used to remove contaminant sequences based on prevalence using decontam in RStudio. The resulting data set was rarefied to a read depth of 750 and subsequently used for analyses of alpha and beta diversity in RStudio.

### Statistical Analysis

To examine differences in microbial communities within host species, we removed *Wolbachia* from the data. *Wolbachia* can account for over 90% of the relative abundance in microbial communities (Ludwick et al., 2019; Paddock et al., 2021), and its association is nearly ubiquitous (Giordano et al., 1997). Conversely, *Wolbachia* infection in NCR populations exhibits strong geographic partitioning (Roehrdanz and Levine, 2007). Analyses conducted with *Wolbachia* can be found in the Supplementary Table S4 and Supplementary Figures S2, S3 Alpha diversity indices (Chao-1 and inverse Simpson’s *D*) for both host species across sites were generated using estimate_richness in the phyloseq package. Analyses of beta-diversity were conducted using a permutational multivariate analysis of variance (PERMANOVA) based on Bray–Curtis and Jaccard distances at the ASV level. Comparisons of differentially abundant taxa present between species were conducted using DESeq2 in RStudio using unrarefied data (Love et al., 2014).

We asked whether the state where a sample was collected from affected microbiome composition within the WCR and NCR. To do this, we analyzed alpha- and beta-diversity separately for each species. For WCR, we also compared microbiome composition across cities within the smaller spatial scale in Colorado and Kansas. Alpha diversity indices (Chao-1 and inverse Simpson’s D) were log-transformed before analysis of variance to correct for non-normal distributions, and significance considered as *p* < 0.05. Analyses of beta-diversity were conducted using a permutational multivariate analysis of variance PERMANOVA based on Bray-Curtis and Jaccard distances at the ASV level. Year of collection was treated as a random factor by restricting permutations within year. Beta dispersion as measured by the average distance to centroid of each group was calculated using vegan::betadisper and compared using a permutational test with vegan::permutest. Pairwise comparisons were made for significant differences observed in PERMANOVA using pairwiseAdonis::pairwiseadonis2 at corrected *p* < 0.05 (Martinez Arbizu, 2020).

To better understand the patterns of community composition at different scales in WCR, we examined the correlation between community dissimilarity and geospatial arrangement. First, we calculated microbiome dissimilarity between individual insects using both Bray-Curtis and Jaccard distances at the ASV level. Geographic distance between sample locations was calculated using the Haversine formula for distance (Robusto, 1957). Resulting distance matrices were analyzed by mantel test using Spearman’s rank-ordered correlation permuted 9,999 times using vegan::mantel. Secondly, values for PC1 for the WCR, individual-species PCoA were extracted using the scores function and then used as the dependent variable in a linear model examining the relationship to geographic distance from a historically relevant origin source region defined as the sample site closest to Mexico along the invasion path (Arizona). To visualize differences between ASVs in WCR collected from different locations, we generated a heat map using the log10 abundance of 125 most abundant ASVs.

### Data Availability

Raw sequences can be found on the NCBI SRA database under the project accession number PRJNA785968. Raw data and accompanying metadata can be found at FigShare at doi: 10.6084/m9.figshare.17130035. Code used in statistical analyses can be found at FigShare at doi: 10.6084/m9.figshare.17130455.

## Results

### WCR and NCR Bacterial Community Composition

**Figure 2:**
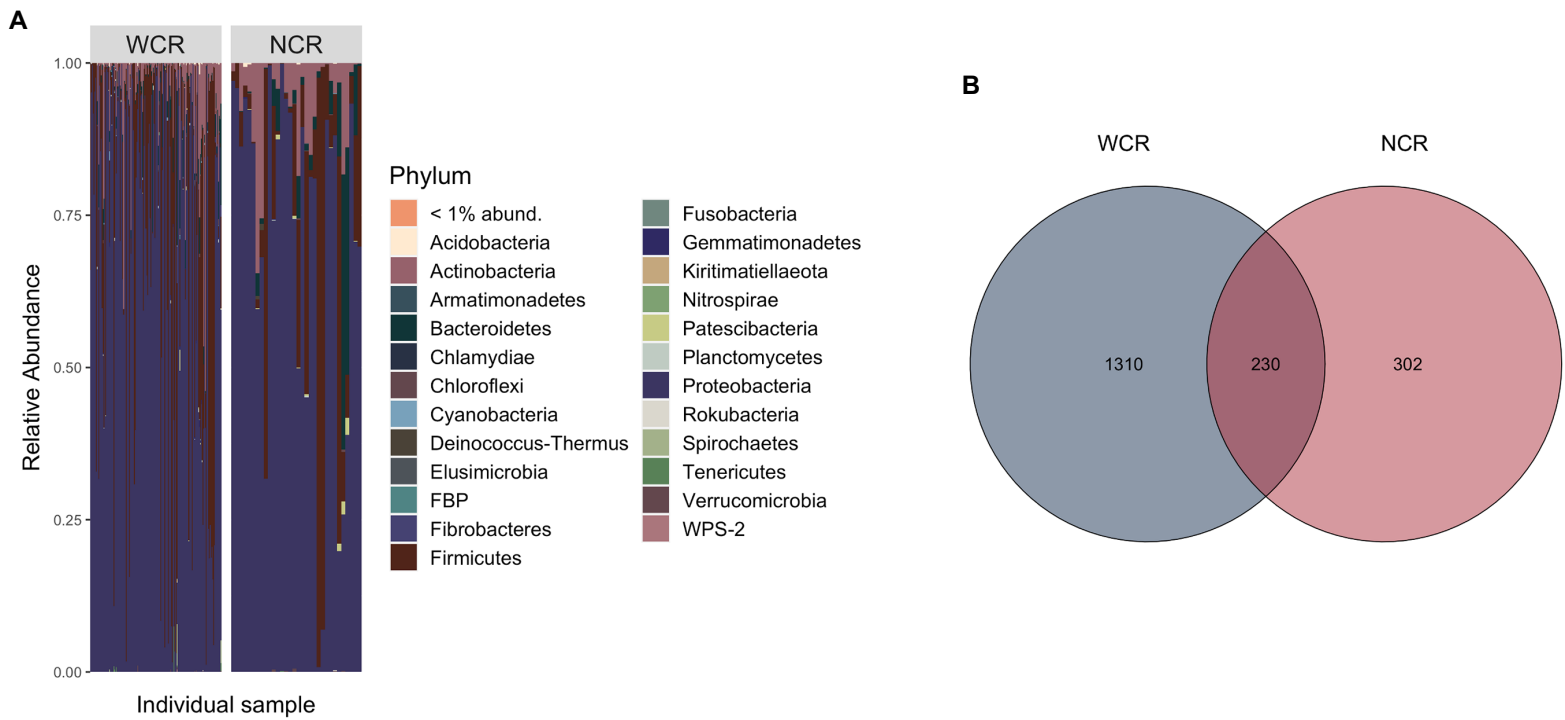
**(A)** Phylum level stacked bar chart of average relative abundance of bacterial communities from adult *Diabrotica virgifera virgifera* (western corn rootworm, WCR) and *Diabrotica barberi* (northern corn rootworm, NCR) collected from across the United States arranged in order of decreasing longitude (west to east). Each bar represents an individual beetle. **(B)** Venn diagram comparing amplicon sequence variant (ASV) overlap in WCR and NCR microbiomes using rarefied data. Data presented with *Wolbachia* removed from communities.

Beetle microbiomes were mainly composed of bacteria from the phyla Proteobacteria, Firmicutes, Actinobacteria, and Bacteriodetes, in order of decreasing relative abundance (Figure 2A). A complete list of all ASVs found within WCR and NCR is located in Supplementary Table 1. Overall, 230 ASVs (~12% of the total ASVs identified) were shared in at least one WCR beetle and one NCR beetle (Figure 2B). These shared ASVs composed 88.2% (±1.5%) of reads in WCR and 91.2% (±3.0%) of NCR reads on average. Shared ASVs can be found in Supplementary Table 2. Seven ASVs were found in each species under a prevalence threshold of 50% presence across all samples per species (Supplementary Table 3). Four of those ASVs were shared between WCR and NCR: two from the family Enterobacteriaceae, and one each from the genera *Pantoea* and *Lactococcus*. ASVs from an unclassified genus in Enterobacteriaceae had the highest relative abundance in both WCR (54.3%) and NCR (40.4%). Other taxa with high relative abundance in WCR were in the genera *Lactococcus*, *Acinetobacter*, *Pseudomonas*, *Serratia*, *Pantoea*, *Microbacterium*, *Massilia*, *Sphingomonas*, and *Exiguobacterium* in order of decreasing relative abundance. In NCR, *Lactococcus*, *Pantoea*, *Serratia*, *Acinetobacter*, *Sphingomonas*, *Pseudomonas*, *Chryseobacterium*, *Stenotrophomonas*, and *Microbacterium* were found in high relative abundance (in decreasing order). Nevertheless, several taxa differed in relative abundance between the two rootworm species (Supplementary Figure 1). Alpha diversity measurements for WCR and NCR were averaged across sites (Table 1).

**Table 1:** Average alpha diversity metrics of western corn rootworm (WCR) and northern corn rootworm (NCR) collected from different locations across the United States.

Species	Chao-1	Inverse Simpson’s D
WCR	34.95 ± 2.25	4.37 ± 0.48
NCR	53.87 ± 6.65	5.06 ± 0.77

### Biogeographical Impact on Bacterial Community Composition

**Figure 3:**
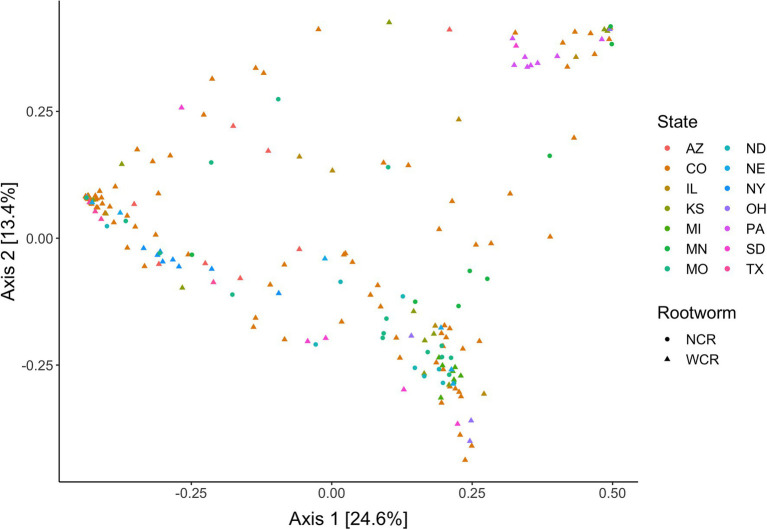
Principal coordinates analysis of bacterial communities in *Diabrotica virgifera virgifera* (WCR) and *Diabrotica barberi* (NCR) collected from their natural host plant corn in the wild based on Bray-Curtis distances. Data presented with *Wolbachia* removed from communities.

**Figure 4:**
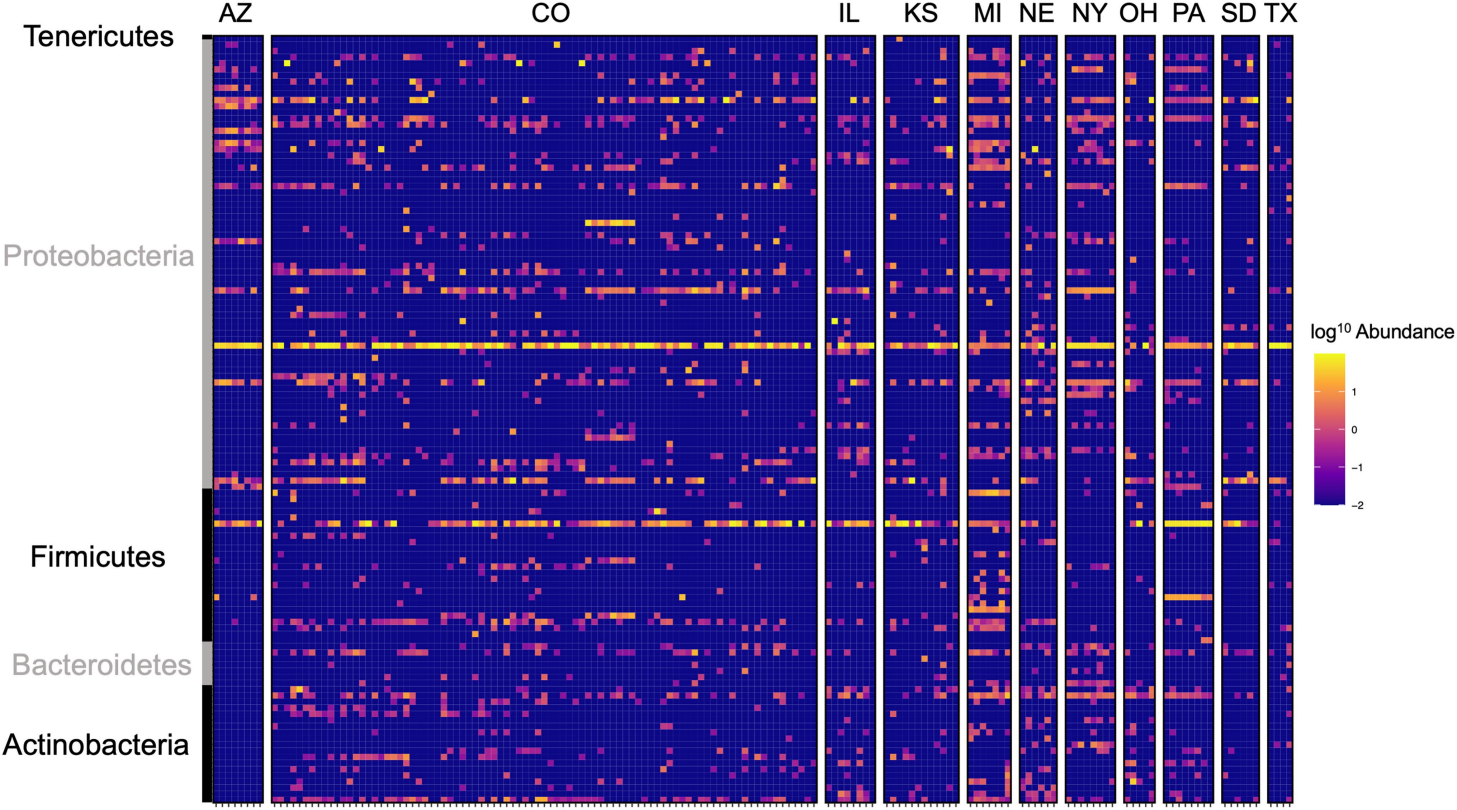
Heat map of log^10^ abundance of the top 125 most abundant ASVs in western corn rootworm (WCR) across sampling locations in the United States. Each row represents a single ASV and each column represents one beetle microbiome collected from one site within the state. Colorado consisted of multiple sampling sites. Family level grouping of ASV are provided on the right of the figure. Data presented with *Wolbachia* removed from communities.

Local environments have an impact on the composition of bacterial communities within WCR and NCR (Figure 3). We found bacterial communities differed at the city and state level in both host species based on the results of the PERMANOVA with Bray-Curtis and Jaccard distances (Table 2). In pairwise comparisons, WCR collected from New York, Pennsylvania, and Michigan consisted of unique bacterial communities different from all other states. Arizona was significantly different from all other locations except Colorado and Illinois. Texas was also different from Kansas and South Dakota. For samples collected from smaller regional-scale sampling scheme in Colorado and Kansas (*n* = 14), overall variation between communities was less than at the landscape level (Table 2). Wild NCR collected from Minnesota harbored unique bacterial communities compared to Missouri and North Dakota. We found no differences between North Dakota and Missouri at the city or state level (Supplementary Table 5). Patterns of differential abundance of ASVs across sites were observed (Figure 4).

**Table 2:** Beta diversity metric comparisons between collection site within host species.

Species	Response	Factor	*df*	*F*	*R* ^2^	*p*
WCR	Community	City	23,158	2.38	0.288	0.001
		State	10,158	3.35	0.184	0.001
		City (Colorado)	13,91	1.45	0.19	0.009
NCR	Community	City	3,31	1.72	0.156	0.002
		State	2,31	2.11	0.127	0.001

Results of models for bacterial community composition (community) in WCR and NCR species collected from differing localities. Distance matrices used in PERMANOVA models were analyzed using Bray-Curtis distances on rarefied data. Western corn rootworm (WCR, Diabrotica virgifera virgifera) and northern corn rootworm (NCR, Diabrotica barberi).

**Figure 5:**
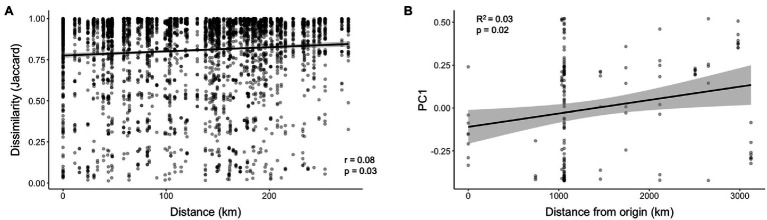
Correlations at various spatial scales between **(A)** Haversine distance and microbiome community dissimilarity (Jaccard) and **(B)** Haversine distance from ancestral population (Arizona) of *Diabrotica virgifera virgifera* (WCR) and values from axis 1 of PCoA of microbiome community similarity based on Jaccard distance.

Pairwise comparisons do not account for geospatial correlation; however, we used mantel tests to investigate correlations between geographic distance and microbiome community dissimilarity between collection locations. The full WCR data set encompassing the continental scale showed no significant correlation between geographic distance and microbiome dissimilarity for Bray-Curtis (*p* = 0.15) or Jaccard (*p* = 0.16) distances. At a smaller geospatial scale (~25 km apart), microbiome dissimilarity increased with increasing distance between sample locations (Figure 5A; Bray-Curtis, *p* = 0.03; Jaccard, *p* = 0.03). We suspected the historical geographic pattern of invasion may be correlated to microbiome similarity. Specifically, we examined the relationship between distance from Arizona, the most ancestral population sampled (Lombaert et al., 2018) and microbiome dissimilarity. The values from PC1 of the PCoA of WCR based on Jaccard distances revealed a significant correlation (Figure 5B; *p* = 0.009). This correlation explained 3% of the variation captured by PC1 (Figure 5B; *R*^2^ = 0.03).

## Discussion

In this study, we found bacterial communities of two insects, WCR and NCR, are significantly impacted by location of collection. However, the geospatial patterning of bacterial communities differed at different spatial scales. The factors governing organization of microbial communities associated with hosts vary in strength across time and space. Populations of WCR can disperse between locations, encountering and mixing microbial populations from isolated patches. If selective environmental forces are similar between locations, the host-associated microbiome may be more affected by dispersal and drift. Thus, community similarity would be expected to decrease over increasing geographical space (Finkel et al., 2012). Here, we found, at smaller spatial scales, bacterial community similarity was correlated with geographic distance between WCR populations. Genetic analyses of the same WCR populations studied here revealed a weak but significant pattern of isolation by distance in Colorado (Kim and Sappington, unpublished data). Our results appear to follow this same pattern of decay by distance (Finkel et al., 2012). We conclude that the spatial turnover observed at the local level in WCR microbiomes is a result of dispersal limitation and ecological drift. However, at broad spatial scales, differences in landscape diversity or host genetics may overshadow dispersal limitation and account for most of the differences in observed microbiome communities separated by long distances.

In *Drosophila*, rapid genomic evolution can occur in response to the microbiome and, at broad spatial scales, differences in microbiome community composition could be driven by local adaptation (Rudman et al., 2019). Previous genetic analysis found a lack of genetic structuring in WCR across most of the United States, presumably because of a lingering lack of genetic equilibrium after the eastward range expansion out of the western Great Plains beginning in the mid-twentieth century (Kim and Sappington, 2005; Flagel et al., 2014). This eastward expansion of WCR (Gray et al., 2009) may also partially explain the lack of distance decay in microbial community composition at the continental scale observed in this study. Populations in New York, Pennsylvania, and Michigan relatively near the eastern front of WCR expansion may exhibit residual effects of founder populations as evidenced by their disparate microbiome communities. Cropping patterns in agricultural landscapes in the eastern United States are more diverse and include less continuously-planted corn than in the central United States, increasing isolation between populations of insects and microbes by hindering dispersal between locations (Onstad et al., 2003). Correlation with distance from a historically relevant origin region (Arizona) for WCR and microbiome composition may reflect an interaction between host genetic structure and isolation by distance. Alternatively, because the agricultural landscapes of the eastern United States differ markedly from those in the Corn Belt, local environmental selection of the microbial species pool could result in significantly different microbial communities found within the insect. It is not well understood what influence the soil microbiome has on adult corn rootworm microbiome composition or which microbes persist through metamorphosis (Ludwick et al., 2019). Feedback between host genetics, the microbial species pool, and dispersal limitation could account for the patterns found here. Further investigation is warranted to elucidate environmental effects (i.e., temperature, landscape composition, and altitude) on host associated microbiomes in rootworms and other insects.

It is possible that differences in microbiome composition derive from management tactics used within a crop field. While the beetles in this study were collected from corn, we do not know what hybrids or management practices were used in the fields of collection. Macro-level changes in diet (i.e., different host plant species) can have pronounced effects on microbiomes across insect species (Jones et al., 2019; Mason et al., 2020), but differences seem to diminish at finer levels of host plant taxonomic resolution (i.e., plant genotypes; Mason et al., 2021). Several corn genotypes harbor unique taxa, but it is unclear how they are affected by their local environment (Favela et al., 2021). Microbiomes of WCR larvae also respond to ingestion of transgenic crops producing *B. thuringiensis* (Bt). Larvae resistant to Bt host a unique microbial community that is less taxonomically rich than susceptible insects (Paddock et al., 2021). We do not know if any populations that we sampled are resistant to Bt. To that end, no studies have investigated whether adult WCR microbiomes also respond to Bt. The functionality of microbial communities in adult WCR are documented to involve adaptation to plant defenses and to influence oviposition sites (Lance, 1992; Chu et al., 2013). However, estimation of individual species functionality in the microbiome of WCR is lacking. Resolution provided by 16S rRNA sequencing does not allow for accurate prediction of functionality and requires further study. While we observed differences between Colorado and other sites, we cannot distinguish the biological signal from a possible kit artifact. In addition, we did not examine microbial strain-level diversity in this study. The increased resolution may illuminate differences in community composition that could not be detected by 16S rRNA sequencing.

Differences in the ecological properties and requirements of microbes may generate variation in the species pool at different locations, which can then affect assembly of host-associated microbiomes. Sample location affects microbiome composition in both species of corn rootworm (Table 1). The local species pool of microbes available to colonize the host likely varies with collection location. For instance, communities within a state are more similar than across states (Table 1), which suggests a local environmental or geospatial effect on bacterial assembly. Microbes associated with a host’s food comprise most of the local species pool for that host (Delsuc et al., 2014; Deb et al., 2019). Thus, specialist insects might have a more predictable microbiome (Gomes et al., 2020). Certain components of corn have a predictable and heritable microbiome (Walters et al., 2018). In our case, WCR adults are likely closely associated with the microorganisms colonizing their diet, sharing taxa found both in corn silk and leaves (Wagner et al., 2020; Khalaf et al., 2021). The most common family found in our samples, Enterobacteriaceae, is generally found in other insects feeding on corn plants as well (Jones et al., 2019), suggesting a close association with corn. Other microbial taxa may be more responsive to abiotic and biotic factors associated with the local environment or may vary in dispersal capacity. Future studies examining the site-specific environmental pool of bacteria would improve the understanding of the impact of food source on microbiome composition in insects.

We found WCR and NCR harbor a small set of commonly occurring bacteria regardless of location. Commonly occurring bacteria (i.e., those represented in 50% of samples) totaled seven for both *D. virgifera virgifera* and *D. barberi*, with four being shared between the two species. This is consistent with other insects which host facultative microbes with high variation between individuals (Blankenchip et al., 2018; Hammer et al., 2020; Silver et al., 2021). The number of shared taxa between WCR and NCR was relatively small (~12%) but accounted for a high amount of the total sequences within the communities (~90%). Specific filtering by host species can structure distinct microbial communities (Jones et al., 2019; Adair et al., 2020), and while we documented distinct taxa within each host species, we cannot be certain they are due to host filtering. A more controlled study is necessary to distinguish environmental and host effects. Still, host species identity does not account for all the interindividual variation observed in host associated microbiomes (Colman et al., 2012; Yun et al., 2014). In our study, the commonly occurring bacteria may capture a large amount of the variation between sites. Consequently, the correlations between locations we observed may be signatures of smaller parts of the bacterial communities found within WCR and NCR, given the effect sizes (Figures 5A,B). Generalist insects may exhibit weaker distance decay patterns due to variation in diet overshadowing local environmental influence.

Integrating landscape ecology, biogeography, and metacommunity theory into host-associated microbiome studies has been complicated. What microbes an animal encounters in its life are influenced in part by the host diet and the environment in which the host interacts (Littleford-Colquhoun et al., 2022). Both the diet and environment are impacted by selective and neutral forces. Host factors such as species identity or life stage also influence microbial community composition (Suárez-Moo et al., 2020). Specialist insects provide a unique opportunity to investigate factors affecting microbiome composition across geographical space. Dispersal limitation between geographically isolated environments results in unique microbial communities. This isolation can also impact host genetic factors that results in variation between microbiomes. Through a metacommunity framework, WCR can be thought of as “islands within islands,” where microorganisms can disperse and establish (or not) on sessile corn plants whereby they interact with another layer of dispersing organisms (insect) that impart their own selective filtering and dispersal barriers onto the microbial communities. This nested structure accounts for variation in dispersal capacity across scales. Understanding how all determinants interact to shape microbiomes within animals is important to better leverage the beneficial effects microbiomes can provide.

## Data Availability Statement

The datasets presented in this study can be found in online repositories. The names of the repository/repositories and accession number(s) can be found in the article/Supplementary Material.

## Author Contributions

All authors conceived of the study and designed the experiments. KP performed the experiments, conducted statistical analysis, and wrote the first draft of the manuscript. KP and DF interpreted the results. All authors contributed to the article and approved the submitted version.

## Conflict of Interest

The authors declare that the research was conducted in the absence of any commercial or financial relationships that could be construed as a potential conflict of interest.

## Publisher’s Note

All claims expressed in this article are solely those of the authors and do not necessarily represent those of their affiliated organizations, or those of the publisher, the editors and the reviewers. Any product that may be evaluated in this article, or claim that may be made by its manufacturer, is not guaranteed or endorsed by the publisher.

## Footnotes

^1^ https://www.qiagen.com/us/resources/resourcedetail?id=00e4513c-597b-4bd5-a600-9259e6d62d07&lang=en

^2^ https://www.qiagen.com/us/resources/resourcedetail?id=68f29296-5a9f-40fa-8b3d-1c148d0b3030&lang=en

^3^ http://journal.embnet.org/index.php/embnetjournal/article/view/200/479
