# Supplementary figures and images for: Patterns of Microbiome Composition Vary Across Spatial Scales in a Specialist Insect
Source: Front Microbiol. 2022 Jun 2;13:898744. doi: 10.3389/fmicb.2022.898744 (PMC9201478; doi:10.3389/fmicb.2022.898744)

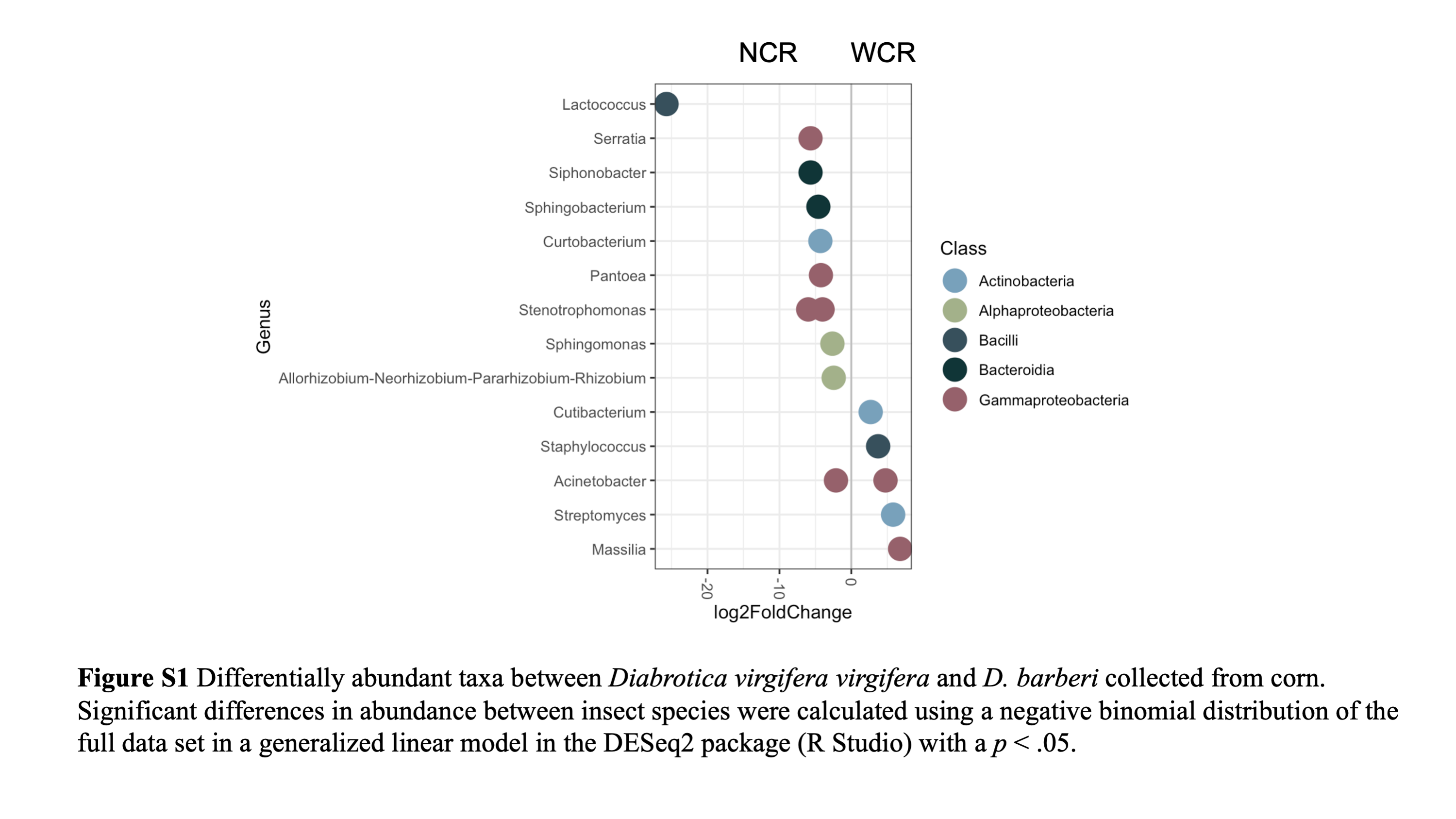

Supplement: Supplementary file 3 [file Image_1.TIFF]

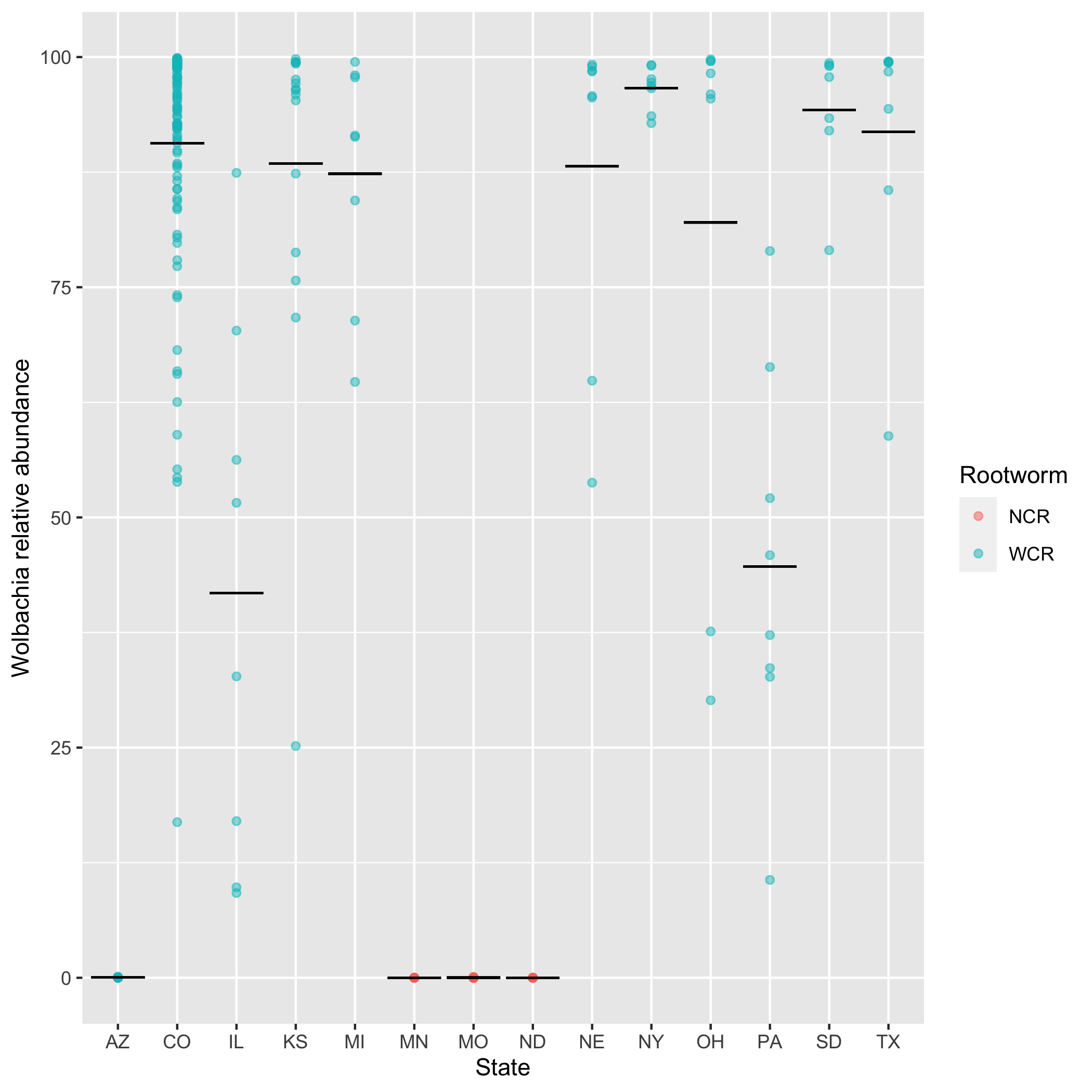

Supplement: Supplementary file 4 [file Image_2.TIFF]

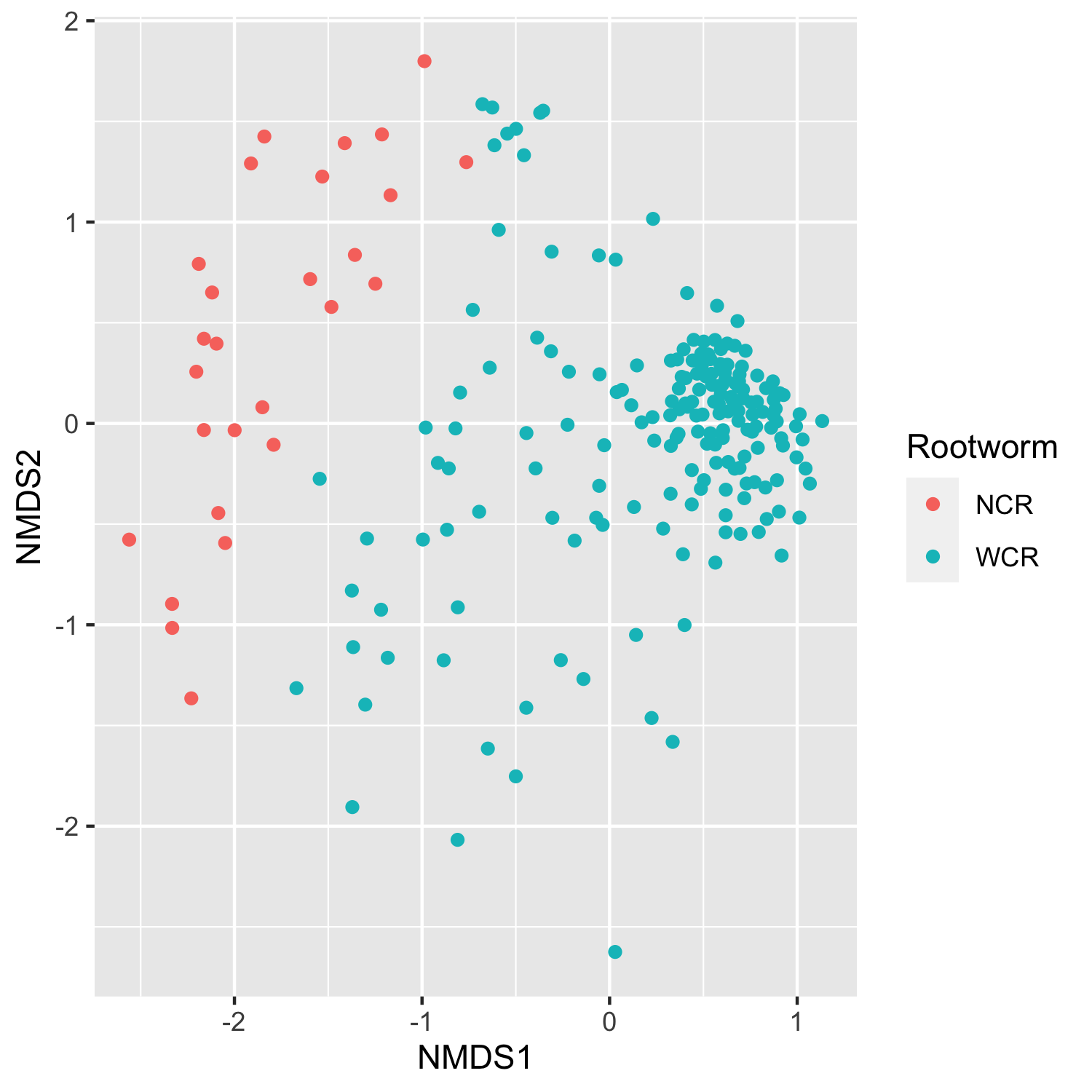

Supplement: Supplementary file 5 [file Image_3.tiff]
